# MicroRNA expression and DNA methylation profiles do not distinguish between primary and recurrent well-differentiated liposarcoma

**DOI:** 10.1371/journal.pone.0228014

**Published:** 2020-01-23

**Authors:** Melissa Vos, Ruben Boers, Anne L. M. Vriends, Joachim Boers, Patricia F. van Kuijk, Winan J. van Houdt, Geert J. L. H. van Leenders, Michal Wagrodzki, Wilfred F. J. van IJcken, Joost Gribnau, Dirk J. Grünhagen, Cornelis Verhoef, Stefan Sleijfer, Erik A. C. Wiemer

**Affiliations:** 1 Department of Medical Oncology, Erasmus MC Cancer Institute, Rotterdam, the Netherlands; 2 Department of Surgical Oncology, Erasmus MC Cancer Institute, Rotterdam, the Netherlands; 3 Department of Developmental Biology, Erasmus University Medical Center, Rotterdam, the Netherlands; 4 Department of Surgical Oncology, Netherlands Cancer Institute, Amsterdam, the Netherlands; 5 Department of Pathology, Erasmus University Medical Center, Rotterdam, the Netherlands; 6 Department of Pathology, Maria Skłodowska-Curie Institute-Oncology Center, Warsaw, Poland; 7 Center for Biomics, Erasmus University Medical Center, Rotterdam, the Netherlands; Chinese University of Hong Kong, HONG KONG

## Abstract

Approximately one-third of the patients with well-differentiated liposarcoma (WDLPS) will develop a local recurrence. Not much is known about the molecular relationship between the primary tumor and the recurrent tumor, which is important to reveal potential drivers of recurrence. Here we investigated the biology of recurrent WDLPS by comparing paired primary and recurrent WDLPS using microRNA profiling and genome-wide DNA methylation analyses. In total, 27 paired primary and recurrent WDLPS formalin-fixed and paraffin-embedded tumor samples were collected. MicroRNA expression profiles were determined using TaqMan^®^ Low Density Array (TLDA) cards. Genome-wide DNA methylation and differentially methylated regions (DMRs) were assessed by methylated DNA sequencing (MeD-seq). A supervised cluster analysis based on differentially expressed microRNAs between paired primary and recurrent WDLPS did not reveal a clear cluster pattern separating the primary from the recurrent tumors. The clustering was also not based on tumor localization, time to recurrence, age or status of the resection margins. Changes in DNA methylation between primary and recurrent tumors were extremely variable, and no consistent DNA methylation changes were found. As a result, a supervised clustering analysis based on DMRs between primary and recurrent tumors did not show a distinct cluster pattern based on any of the features. Subgroup analysis for tumors localized in the extremity or the retroperitoneum also did not yield a clear distinction between primary and recurrent WDLPS samples. In conclusion, microRNA expression profiles and DNA methylation profiles do not distinguish between primary and recurrent WDLPS and no putative common drivers could be identified.

## Introduction

Soft tissue sarcomas form a heterogeneous group of rare, mesenchymal tumors, of which liposarcomas comprise one of the largest subgroups.[1] Of all 100–120 patients diagnosed annually with liposarcoma in the Netherlands[2], the most common subtype is well-differentiated liposarcoma (WDLPS). WDLPS are mostly localized in the extremities and the retroperitoneum, and the prognosis of these patients is significantly better than those of patients with dedifferentiated liposarcoma.[2] However, WDLPS have a risk of dedifferentiation, potentially leading to metastatic disease with concurrent dismal prognosis. The rate of dedifferentiation in WDLPS in the extremities is extremely low, while in the retroperitoneum the risk of dedifferentiation is higher.[1] Molecularly, WDLPS are characterized by amplification–on a neochromosome–of the 12q14-15 region, which includes the genes *MDM2* and *CDK4*.[1] Treatment of WDLPS consists of complete surgical resection of the tumor, occasionally combined with neoadjuvant/adjuvant radiotherapy for tumors localized in the retroperitoneum. Unfortunately, approximately one-third of the patients will develop a local recurrence. Whereas the biology and behavior of primary WDLPS has been widely studied, there is a lack of insight in changes in microRNA expression and DNA methylation profiles between primary and recurrent WDLPS.

MicroRNAs have been proven to play a significant role in tumorigenesis[3–5], including in soft tissue sarcomas and more specifically liposarcomas.[6–11] So far, microRNA expression profiles have been used to differentiate between different liposarcoma subtypes[6–9, 12, 13] or to predict patient outcome.[10, 11, 14, 15] However, it is unclear whether primary WDLPS and their recurrent tumors can be distinguished by their microRNA profiles, which would suggest that microRNAs may be involved in the process of recurrence.

DNA methylation is an epigenetic process that fulfils an essential role in physiological and biological processes[16], and can be an important pathological driver in cancer.[17, 18] DNA methylation patterns can be utilized as biomarker[19, 20], to classify cancer (sub)types[21, 22] or to predict outcome.[20, 23] Genome-wide DNA methylation analysis used to be technically challenging and costly, but recently a new method was developed showing accurate genome-wide analysis of CpG-methylation by using the DNA methylation-dependent restriction enzyme *LpnPI* and subsequent DNA sequencing of the restriction fragments.[24] This methylated DNA sequencing (MeD-seq) technology is cost-effective, accurate and reproducible with high coverage, suitable for high-throughput epigenetic profiling, even on FFPE material. For liposarcoma in general and recurrent WDLPS specifically, the knowledge of epigenetics is limited. Only a few studies report on the role of DNA methylation in liposarcomas, but mostly focus on one specific DNA region in more aggressive liposarcomas subtypes.[25, 26] Some studies report a link between DNA methylation and microRNAs, for example methylation-induced silencing of miR-193b in dedifferentiated liposarcoma but not in WDLPS[27] and low expression of miR-193b, due to downregulation by promoter methylation, resulting at least partly from an increased expression of DNA methyltransferase-1.[28]

In this study, we molecularly compared primary and recurrent WDLPS at microRNA and DNA methylation level aiming to discover differences and/or similarities that give insight in the biology of recurrent WDLPS.

## Materials and methods

### Patients and samples

Patients with available tumor samples of a primary and matching first recurrent WDLPS who were treated with surgery only were included. The formalin-fixed and paraffin-embedded (FFPE) tissue blocks were obtained through PALGA, the Dutch nationwide pathology registry, and the pathology department of the Maria Skłodowska-Curie Institute-Oncology Center together with anonymized clinicopathological information. The resection margins were defined as R0 (microscopically negative margins), R1 (microscopically positive margins), R2 (macroscopically positive margins) or Rx (unknown/not assessed). Although recurrence after R1/R2 resections can be considered as progressed WDLPS rather than truly recurrent WDLPS, these will be referred to as recurrent WDLPS as well. To calculate time to recurrence, the resection dates stated in the pathology reports were used. Each pair received an individual number with index numbers designating the primary tumor (.1) or recurrent tumor (.2).

The experimental protocol was reviewed and approved by the Medical Ethics Committee of the Erasmus MC (MEC-2016-213). All experimental procedures were performed in accordance with the relevant guidelines and regulations, including the Helsinki Declaration. The use of anonymous or coded left-over material for scientific purposes is part of the standard treatment agreement with patients and therefore additional informed consent was not asked.

### RNA and DNA isolation

The archival tumor samples were examined by an expert pathologist to confirm the initial histopathological diagnosis and to determine the percentage of tumor cells. The diagnosis of WDLPS was based either on the presence of lipomatous cells with fibrous septa and spindle cells with hyperchromatic irregular nuclei, or on the amplification of the MDM2-gen using FISH in case morphological atypia was less conspicuous. Only sections containing approximately 100% tumor cells were used for isolation. Total RNA was isolated using the RecoverAll^™^ Total Nucleic Acid Isolation Kit (Ambion/Life Technologies) and total DNA was isolated using the AllPrep^®^ DNA/RNA FFPE kit (Qiagen), both according to manufacturer's instructions.

### MicroRNA expression profiling

MicroRNA expression was determined using TaqMan^®^ Low Density Array (TLDA) cards (A card v2.0, B card v3.0, Applied Biosystems/Thermo Fisher Scientific). Megaplex^™^ RT Primers (Human Pool, pool A v2.1, pool B v3.0, Applied Biosystems/Thermo Fisher Scientific) were used for cDNA synthesis, followed by a standard pre-amplification protocol using Megaplex^™^ PreAmp Primers (Human Pool, pool A v2.1, pool B v3.0, Applied Biosystems/Thermo Fisher Scientific). The TLDA cards were analysed using a 7900HT Real-Time PCR system (Applied Biosystems). The paired samples were processed in three batches for logistical and technical reasons, with each primary and its matching recurrent tumor being placed within the same batch.

### Statistical analysis of microRNA profiling data

The expression of each microRNA in a sample was normalized to the median Ct-value of all detectable microRNAs in that sample. The normalized relative expression was subsequently calculated for each microRNA and log-transformed. Since the samples were processed in multiple batches, potential batch-effects were investigated using PCA-plots in R (S1 Fig). To correct for the observed batch-effects, ComBat was used[29]. Only microRNAs detected in at least 50% of the samples were included in the statistical analyses. A paired t-test was performed to identify microRNAs that were differentially expressed between paired primary and recurrent WDLPS samples. A two-sided p-value <0.05 was considered statistically significant. To adjust for multiple testing, a false discovery rate (FDR) of 0.25 was used. For all microRNA clustering analyses, the software program Cluster 3.0 was used followed by Java TreeView for visualization of the clustering results. The microRNA expression datasets generated and analysed during the current study have been deposited to the Gene Expression Omnibus (GEO) data repository under submission number GSE137722.

### MeD-seq sample preparations

MeD-seq analyses were essentially carried out as previously described[24]. DNA samples were digested by LpnPI (New England Biolabs). Stem-loop adapters were blunt-end ligated to repaired input DNA and amplified to include dual indexed barcodes using a high fidelity polymerase to generate an indexed Illumina NGS library. The amplified end product was purified on a Pippin HT system with 3% agarose gel cassettes (Sage Science). Multiplexed samples were sequenced on Illumina HiSeq2500 systems for single reads of 50bp according to manufacturer’s instructions. Dual indexed samples were demultiplexed using bcl2fastq software (Illumina).

### MeD-seq data analysis

Data processing was carried out using specifically created scripts in Python. Raw fastq files were subjected to Illumina adaptor trimming and reads were filtered based on LpnPI restriction site occurrence between 13-17bp from either 5’ or 3’ end of the read and mapped to hg38 using bowtie2. Genome-wide individual LpnPI site scores were used to generate read count scores for the following annotated regions (www.ensembl.org): transcription start sites (TSS, 1 kb before and 1 kb after), CpG-islands and gene bodies (1kb after TSS till TES). Detection of differentially methylated regions (DMRs) was performed between two datasets using the χ2-test on read counts. Significance was called by either Bonferroni or FDR using the Benjamini-Hochberg procedure.

In addition, a genome-wide sliding window was used to detect sequentially differentially methylated *LpnPI* sites. Statistical significance was called between *LpnPI* sites in predetermined groups using the χ2-test. Neighboring significantly called *LpnPI* sites were binned and reported. Annotation of the overlap was reported for TSS, CpG-islands and gene body regions. DMR thresholds were based on *LpnPI* site count, DMR sizes (in bp) and fold changes of read counts as mentioned in the figure legends before performing hierarchical clustering. The differentially methylated datasets generated and analyzed during the current study have been deposited to the Sequence Read Archive (SRA) under submission number PRJNA574561.

## Results

### Patient samples

In total 27 pairs of patient samples were collected: 16 from the Erasmus MC Cancer Institute, 9 from the Netherlands Cancer Institute, and 2 from the Maria Skłodowska-Curie Institute-Oncology Center. The extremity was the most common localization (N = 15), followed by the retroperitoneum (N = 8). Fourteen patients were female, 13 patients were male. The median age at time of diagnosis of the primary tumor was 59 years (interquartile range [IQR] 50–64) and the median time to recurrence was 3.7 years (IQR 1.9–6.5). In a number of patients (N = 8, 29.6%), the status of the resection margins of the primary tumor was unknown, not assessed or not specified (Rx) in the pathology report. Of those patients of whom the status of the resection margins was reported, all primary resections were R0 or R1 resections, except for one patient (no. 17) with tumor localization in the esophagus, who underwent a R2 resection. Resections of the recurrent tumors resulted in 4 patients in R2 resections (Table 1).

**Table 1:** Patient and tumor characteristics.

Sample	Age^†^	Sex	Localization	Resection margins	Time to recurrence^‡^	No. of DMRs
1.1	64	Female	Upper leg	R1	3.7	32,854
1.2	68			R1		
2.1	78	Male	Retroperitoneal	R1	1.9	2,430
2.2	79			R2		
3.1	58	Female	Upper leg	R1	10.6	4,410
3.2	69			R1		
4.1	50	Male	Upper leg	Rx	8.3	1,061
4.2	59			R2		
5.1	62	Male	Axilla	R0	5.3	1,191
5.2	67			Rx		
6.1	31	Female	Upper leg	R1	8.5	2,732
6.2	39			R0		
8.1	60	Male	Lower leg	Rx	1.0	724
8.2	61			Rx		
9.1	38	Female	Upper leg	R1	2.1	675
9.2	40			R1		
10.1	68	Female	Mediastinum	R0	1.3	1,747
10.2	69			R1		
11.1	52	Female	Retroperitoneal	Rx	2.6	1,028
11.2	54			Rx		
13.1	50	Female	Retroperitoneal	R1	8.1	3,659
13.2	58			R1		
14.1	64	Male	Upper leg	R0	0.6	636
14.2	64			R1		
15.1	55	Female	Retroperitoneal	R1	2.0	1,920
15.2	57			R1		
16.1	48	Male	Lower leg	R1	0.4	473
16.2	48			R1		
17.1	70	Male	Esophagus	R2	0.1	586
17.2	70			R2		
19.1	43	Male	Upper leg	R1	4.7	7,644
19.2	48			R1		
20.1	64	Male	Upper leg	R1	6.5	21,585
20.2	70			R1		
21.1	52	Male	Retroperitoneal	R0	3.5	1,481
21.2	56			R0		
22.1	59	Female	Retroperitoneal	Rx	4.2	314
22.2	63			R1		
23.1	47	Male	Upper leg	R1	16.6	1,119
23.2	63			R0		
24.1	76	Female	Upper leg	R1	3.0	372
24.2	79			R0		
25.1	49	Female	Upper leg	Rx	3.9	482
25.2	53			R1		
26.1	50	Female	Retroperitoneal	Rx	2.1	2,513
26.2	53			Rx		
27.1	60	Male	Retroperitoneal	Rx	1.5	1,377
27.2	61			R1		
28.1	71	Female	Upper leg	R0	6.1	1,910
28.2	77			R1		
29.1	60	Male	Trunk	Rx	13.8	2,819
29.2	74			R1		
30.1	61	Female	Upper leg	R1	4.6	294
30.2	66			R2		

DMR: differentially methylated region.

^**†**^Age at time of surgery.

^‡^in years

### MicroRNA profiling of paired primary–recurrent WDLPS samples

**Fig 1:**
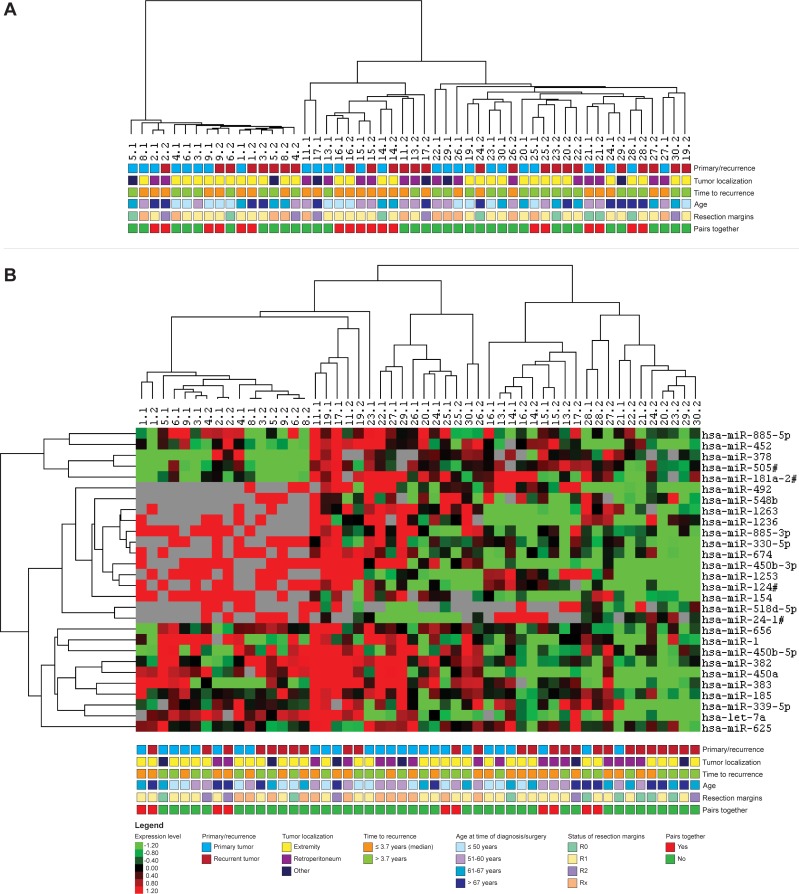
Hierarchical clustering based on the microRNA expression levels of 26 paired primary and recurrent WDLPS tumor samples. (**A**) Results of an unsupervised clustering analysis, depicted with time to recurrence, tumor localization, age and the status of the resection margins. Tumor pairs that cluster together in the same branch of the cluster tree are indicated with red boxes in the bottom line of the figure. (**B**) Results of a supervised clustering analysis based on the expression of 28 significant differentially expressed microRNAs (p<0.05, FDR<0.25), together with time to recurrence, tumor localization, age, the status of the resection margins and an indication of primary–recurrent pairs that cluster together. Grey designates missing expression values.

After correction for batch effect, samples 10.1 and 10.2 were excluded from further microRNA analyses (S1 Fig). First, an unsupervised hierarchical clustering analysis was performed to group the samples based on their microRNA expression profiles without prior knowledge of the origin of the sample (primary or recurrent). This clustering did not show a clear distinction between primary and recurrent WDLPS samples, neither a discriminative pattern based on tumor localization, time to recurrence, age nor the status of the resection margins could be observed (Fig 1A). In 9 of the 26 pairs, the primary and recurrent tumor samples clustered together (indicated by the red squares in the bottom row of the figure). All of these pairs had a short time to recurrence (before the median time to recurrence of 3.7 years), except one pair with a time to recurrence of 3.9 years and one pair with a time to recurrence of 6.1 years.

Next, a supervised analysis was performed based on the expression levels of the 28 significant differentially expressed microRNAs (p<0.05, FDR<0.25)(Fig 1B, S1 Table). The heat map indicated no clear discriminative pattern between primary and recurrent WDLPS, nor a distinction based on tumor localization, time to recurrence, age or the status of the resection margins. Five pairs clustered together, but clustering of these pairs also did not seem to be driven by one of the clinicopathological parameters.

**Fig 2:**
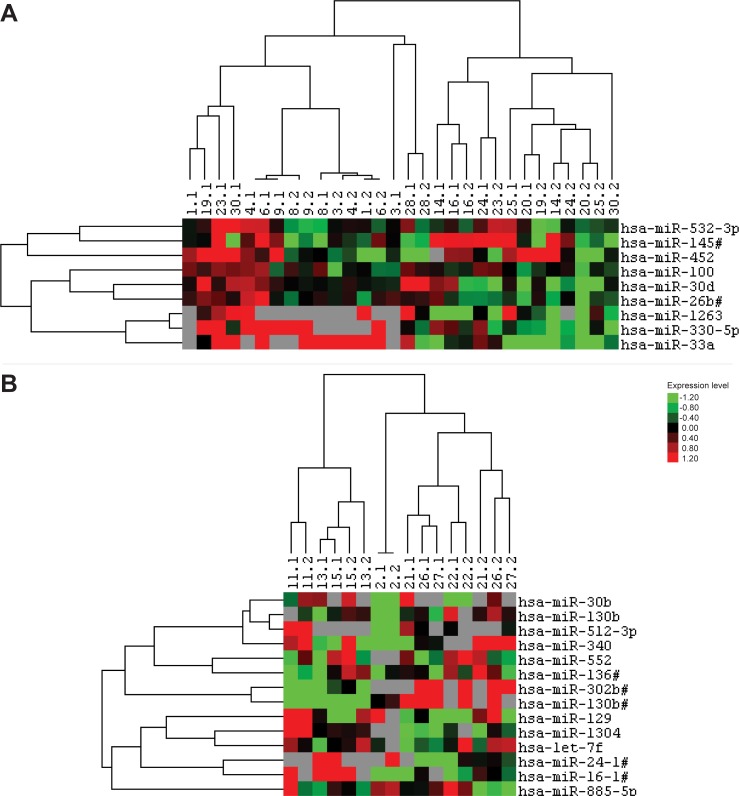
Hierarchical clustering based on the microRNA expression levels of paired primary and recurrent WDLPS tumor samples of the two main tumor localizations. Grey designates missing expression values. (**A**) Results of a supervised clustering analysis based on nine differentially expressed microRNAs (p<0.05, FDR<0.25; N = 15 pairs) between primary and recurrent WDLPS of the extremity. (**B**) Results of a supervised clustering analysis based on 14 differentially expressed microRNAs (p<0.05; N = 8 pairs) between primary and recurrent WDLPS of the retroperitoneum.

Since microRNA expression is reported to be (partially) tissue specific[30], it may be influenced by the localization of the tumor. Therefore, additional sub-analyses for the two largest subgroups regarding tumor localization were performed: the extremity (N = 15 pairs, Fig 2A) and the retroperitoneum (N = 8 pairs, Fig 2B). For the tumor samples localized in the extremity, 68 microRNAs were significantly differentially expressed between primary and recurrent WDLPS of which 9 had an FDR<0.25 (Fig 2A, S2 Table). A cluster analysis based on the expression of these microRNAs did not seem to depend on primary/recurrence, time to recurrence, age or status of the resection margins. For the retroperitoneal WDLPS, only 14 microRNAs were significantly differentially expressed, of which none had an FDR<0.25 (S2 Table). Therefore, the microRNAs with p<0.05 without FDR correction were used to generate a heat map for this subgroup (Fig 2B). Again, no distinction between primary and recurrent samples was observed.

### DNA methylation patterns of paired primary–recurrent WDLPS samples

**Fig 3:**
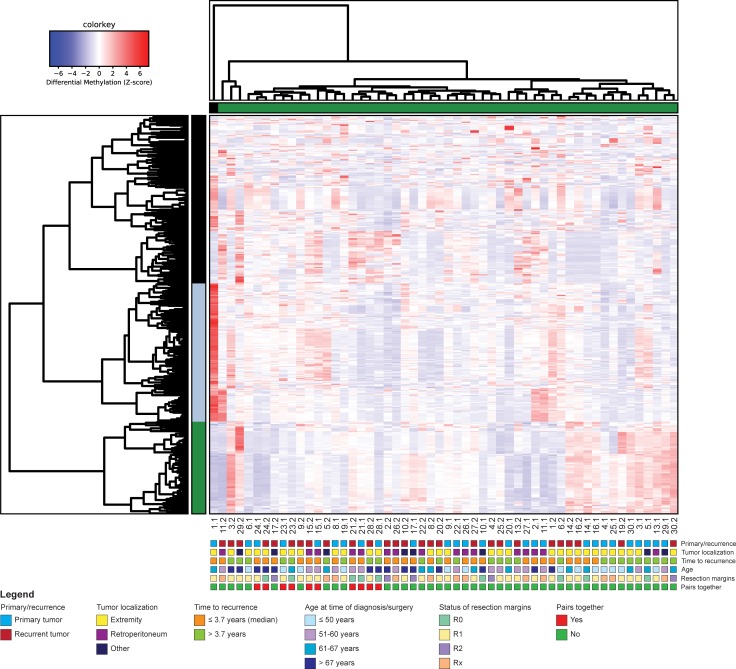
Hierarchical clustering based on differentially methylated DNA regions (DMRs) between primary and recurrent WDLPS samples. The heat map depicts a supervised clustering of the 27 paired WDLPS samples based on 455 differentially methylated regions (DMRs), excluding sex chromosomal regions (N = 15 DMRs), together with the clinicopathological features time to recurrence, tumor localization, age and the status of the resection margins.

When comparing differentially methylated DNA regions (DMRs) between individual primary and recurrent WDLPS pairs, it was noted that the DNA methylation differences were extremely variable between pairs (Table 1), although most of the pairs with a short time to recurrence (before median time to recurrence of 3.7 years) tended to have a lower number of DMRs. However, samples with a longer time to recurrence, for example sample pairs 23 and 28, also displayed relative low numbers of DMRs, and sample pair 1, which had a short time to recurrence, exhibited the largest number of DMRs (Table 1). These DNA methylation differences seemed to be inconsistent among the individual pairs and could not be identified when comparing primary tumors versus recurrent tumors as a group. In the total group, only a relatively small number of 470 DMRs were identified, located on various chromosomes (S3 Table). When these DMRs were used for a supervised hierarchical clustering analysis, no clear clustering of the 27 primary and recurrent samples was observed (Fig 3). Likewise, no distinction was detected based on the clinicopathological parameters (Fig 3). Five of the pairs clustered together, but again across these samples no similarities in terms of time to recurrence, localization, or the status of the resection margins could be identified.

A relatively high number of the observed 470 DMRs was located at chromosome 12 (S3 Table), including DMRs linked to the genes *MDM2*, *CDK4* and *MIR26A* (S4 Table). These DMRs might indicate a possible difference in methylation of (regions of) chromosome 12 between primary and recurrent WDLPS, albeit the fold changes between the groups are relatively low (S4 Table). The highest fold change observed was 2.03 for the gene *RP11-611E13*.*2*, a relatively unknown gene located on chr12q15, the same region as *MDM2*, encoding a non-coding RNA. For *MDM2*, which is amplified in WDLPS, eight DMRs were found, with a fold change of 1.29 for the highest DMR.

**Fig 4:**
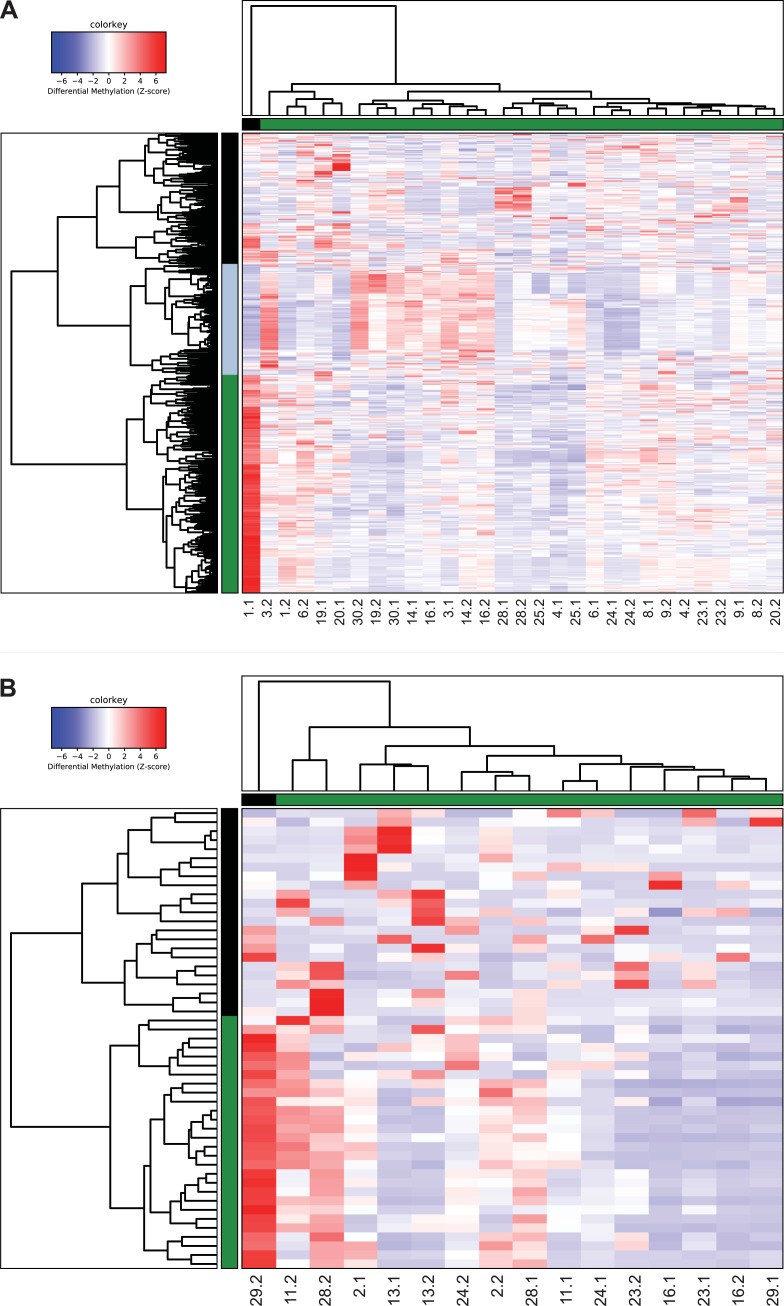
Hierarchical clustering based on differentially methylated DNA regions (DMRs) between paired WDLPS tumor samples for the two main localizations. (**A**) Results of the hierarchical supervised clustering, excluding sex chromosomal regions (N = 27), based on 604 DMRs of the 15 paired WDLPS samples localized in the extremity. (**B**) Results of the hierarchical supervised clustering analysis based on the 51 DMRs with a fold change ≥2, excluding sex chromosomal regions (N = 2), of the 8 paired retroperitoneal WDLPS samples.

Since DNA methylation patterns are also tissue-specific[24, 31, 32] and may be affected by tumor localization, subgroup analyses for the two main localizations were performed: the extremity (N = 15 pairs) and the retroperitoneum (N = 8 pairs). For the tumor samples located in the extremity, 631 DMRs were identified between primary and recurrent samples. Also here, no clear clustering pattern could be identified based on primary/recurrent WDLPS, time to recurrence or the status of the resection margins (Fig 4A). For the tumor samples localized in the retroperitoneum, 1,071 DMRs were identified. To prevent the clustering from being blurred by background noise due to the higher number of DMRs, the clustering analysis for the retroperitoneal tumors was based on the DMRs with a fold change >2 (N = 53 DMRs). Again, this did not lead to a clear distinction between primary and recurrent WDLPS samples (Fig 4B).

## Discussion

To the best of our knowledge, this is the first paper comparing paired primary WDLPS samples to recurrent WDLPS samples at a molecular level. We aimed to gain more insight into the biology of (recurrent) WDLPS and thereby the process of recurrence. The finding that no clear distinction could be made between primary and recurrent WDPLS based on differentially expressed microRNAs or differentially methylated DNA regions suggests that there are no common alterations or that the alterations in microRNA expression and DNA methylation are very heterogeneous and variable between individual patients.

In the unsupervised microRNA clustering analysis, 7 of the 13 pairs (54%) with a short time to recurrence (before median time to recurrence) clustered together, compared to 2 of the 13 pairs (15%) with a longer time to recurrence. This might point towards a recurrence through the outgrowth of a residue in these patients, rather than a recurrence that originates from a single tumor cell. Alternatively, it might suggest that early recurrent tumors resemble each other more closely than late recurrent tumors, because they have had less time to change.

Of the 28 differentially expressed microRNAs, miR-1263 was the most significant differentially expressed microRNA, a relatively unknown microRNA whose role in cancer has not been established yet, followed by miR-885-5p. Upregulation of this microRNA has been linked to enhanced proliferation and migration[33], and the development of liver and lung metastases in colorectal cancer.[34] In contrast, miR-885-5p suppressed proliferation, migration and invasion *in vitro* in osteosarcoma cells, and was downregulated in osteosarcoma patients with low expression levels being associated with a poor prognosis.[35] In our study, miR-885-5p was downregulated in the recurrent tumors, possibly matching the findings in osteosarcoma with low levels of miR-885-5p being associated with more proliferation and a poorer prognosis. Lastly, in our comparison of primary and recurrent WDLPS we did not detect differential expression of the microRNAs that were previously found to be important for sarcomagenesis in WDLPS, such as miR-628[6], miR-675[6], miR-26a[8], miR-451[8] or miR-193b.[28] However, these microRNAs were all discovered in comparisons with 'normal' fat tissue.

**Fig 5:**
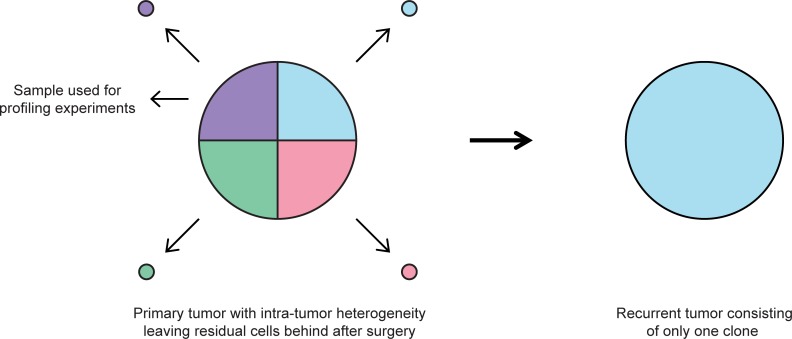
Schematic overview of the concept of intra-tumor heterogeneity in the context of the current study. If the primary tumor sample that was used for the experiments mainly consists of one specific cancer cell subtype, but the recurrent tumor is a recurrence of mainly other cancer cell subtypes, this might explain the large variability in DNA methylation and microRNA expression, even in case of short time to recurrence.

Remarkably, only 470 DMRs with relatively low fold changes were identified between primary and recurrent WDLPS, which is a relatively small number considering the thousands of potential DNA methylation sites in the genome. Possibly, this can be explained by the low-grade nature of this tumor type.[1] Furthermore, there was large variability in the number of DMRs between the pairs, ranging from 294 to 32,854 DMRs. Given our extensive efforts to compose a homogenous dataset by selecting only WDLPS without any neoadjuvant/adjuvant treatment and using only sections almost entirely consisting of tumor tissue, it seems that the inter-tumor heterogeneity is abundant. This heterogeneity–in DNA methylation as well as in microRNA expression–could also be due to intra-tumor heterogeneity, such as exists in other cancers. The concept of intra-tumor heterogeneity describes the observation that a tumor may exist of different tumor cells with distinct molecular and genomic profiles. If the used primary tumor sample was taken of one part of the tumor, but the recurrence mainly consists of cells from another part of the tumor or of cells that had a relatively small contribution to the primary tumor, this might explain the differences in microRNA expression profiles and DNA methylation patterns, even in case of a short time to recurrence (Fig 5). However, currently it is unknown whether such an intra-tumor heterogeneity is present in WDLPS.

A relatively high number of DMRs occurred in chromosome 12, including DMRs linked to *MDM2*, suggesting that hypermethylation of chromosome 12 plays a role in recurrence. However, with the MeD-seq method one cannot reliably discriminate copy number variations from actual differences in DNA methylation. Since WDLPS is characterized by amplification of a specific region on chromosome 12 (12q14-15)[1, 36], including *MDM2* and *CDK4* amongst others, we cannot reliably distinguish between additional amplification or actual changes in DNA methylation.

A limitation of the study was that in approximately a third of the patients the status of the resection margins of the primary surgery was not specified in the pathology report. Unfortunately, due to the retrospective nature of the study, which is inevitable when studying extremely rare diseases like WDLPS, we were not able to retrieve these. However, this percentage (29.6%) of missing resection margins is not unusual and in line with the number (24.0%) of pathology reports lacking information on the resection margins in a nationwide study on sarcoma care in the Netherlands.[37] The strengths of this study were the relatively large sample size and the use of paired samples collected from multiple centers. Both microRNAs and DNA methylation are known to vary–to a certain extent–between individuals[38, 39], and by using paired samples, we aimed to eliminate or minimize this inter-individual variability, so that only microRNAs and DMRs involved in sarcomagenesis would remain in the analyses.

The results of this study suggest that there are no common alterations on microRNA or DNA methylation level that are possibly involved as drivers in the process of recurrence. The next question is whether recurrent WDLPS has different molecular abnormalities upfront, i.e. in the primary tumor, than those who do not recur. Therefore, for a future research project we would recommend to compare primary WDLPS samples of patients who did not develop a recurrence to primary WDLPS tumor samples of patients who did develop a recurrence.

## Conclusion

Primary and recurrent WDLPS cannot be distinguished based on microRNA expression profiles and DNA methylation patterns. Although no common alterations for recurrence could be revealed, a role for microRNAs and DNA methylation in the process of recurrence cannot be ruled out completely, since the aberrations contributing to recurrence might be very heterogeneous and variable between individuals. Alternatively, other molecular events may underlie WDLPS recurrence.

## Supporting information

S1 Fig Visualization of principal component analyses (PCA) using the microRNA expression data as input. The panels depict the PCA before (**A**) and after (**B**) correction for batch effects. Based on the analyses shown in panel B, data from sample 10.1 and 10.2 were excluded from further microRNA analyses, resulting in the PCA analysis in the third panel (**C**).
(PDF)

S1 Table Differentially expressed microRNAs. All differentially expressed microRNAs (p<0.05, FDR<0.25, N = 28 microRNAs) between primary and recurrent WDLPS of 26 paired tumor samples.
(PDF)

S2 Table Differentially expressed microRNAs in subgroup analyses of the extremity and retroperitoneum. All differentially expressed microRNAs between 15 paired primary and recurrent WDLPS tumor samples of the extremity (p<0.05, FDR<0.25, N = 9 microRNAs) (**A**) and of the 8 paired primary and recurrent WDLPS tumor samples of the retroperitoneum (p<0.05, no FDR, N = 14 microRNAs)(**B**).
(PDF)

S3 Table List of the numbers of DMRs per chromosome. (PDF)

S4 Table Top 100 genes with a DMR. Top 100 genes that contain at least one differentially methylated DNA region (DMR) after Bonferroni correction, excluding genes/DMRs located on the sex chromosomes, found by MeD-seq on 27 paired primary and recurrent WDLPS tumor samples.
(PDF)
