# Supplementary material for: MicroRNA expression and DNA methylation profiles do not distinguish between primary and recurrent well-differentiated liposarcoma
Source: PLoS One. 2020 Jan 23;15(1):e0228014. doi: 10.1371/journal.pone.0228014 (PMC6977735; doi:10.1371/journal.pone.0228014)

S1 Fig. Visualization of principal component analyses (PCA) using the microRNA expression data as input. The panels depict the PCA before (**A**) and after (**B**) correction for batch effects. Based on the analyses shown in panel B, data from sample 10.1 and 10.2 were excluded from further microRNA analyses, resulting in the PCA analysis in the third panel (**C**).

A

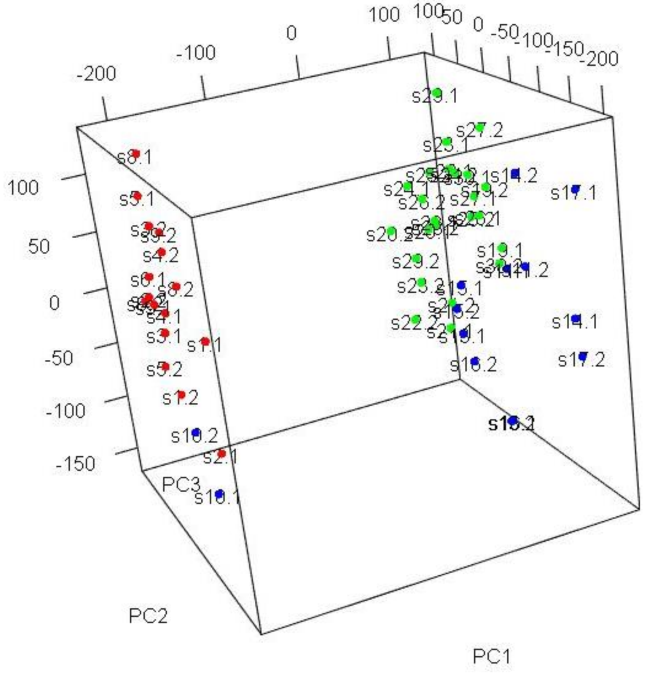

B

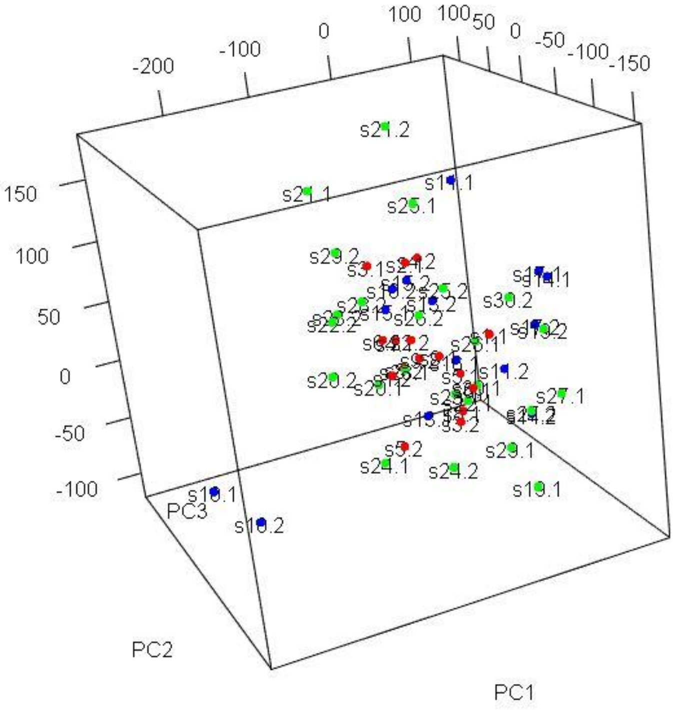

C

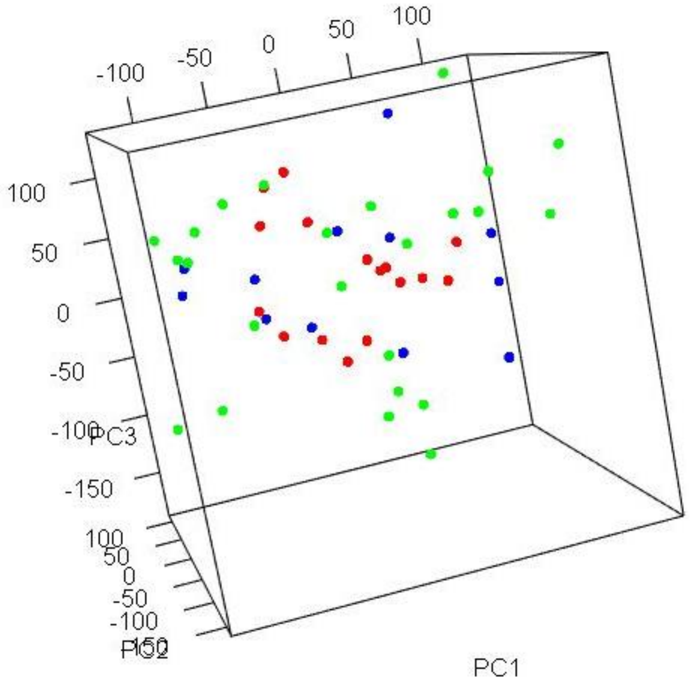

Supplement: S1 Fig — The panels depict the PCA before (A) and after (B) correction for batch effects. Based on the analyses shown in panel B, data from sample 10.1 and 10.2 were excluded from further microRNA analyses, resulting in the PCA analysis in the third panel (C). (PDF) [file pone.0228014.s001.pdf]
