# Supplementary material for: MicroRNA expression and DNA methylation profiles do not distinguish between primary and recurrent well-differentiated liposarcoma
Source: PLoS One. 2020 Jan 23;15(1):e0228014. doi: 10.1371/journal.pone.0228014 (PMC6977735; doi:10.1371/journal.pone.0228014)
Supplement: S1 Table — All differentially expressed microRNAs (p<0.05, FDR<0.25, N = 28 microRNAs) between primary and recurrent WDLPS of 26 paired tumor samples. (PDF) [file pone.0228014.s002.pdf]

S1 Table. Differentially expressed microRNAs. All differentially expressed microRNAs (p<0.05, FDR<0.25, N=28 microRNAs) between primary and recurrent WDLPS of 26 paired tumor samples.

microRNA	Upregulated in	Fold change	% detection	p-value	FDR
hsa-miR-1263	Primary	1.209	73%	0.0001	0.041
hsa-miR-885-5p	Primary	1.987	100%	0.0006	0.132
hsa-miR-885-3p	Primary	4.699	87%	0.0010	0.132
hsa-miR-656	Primary	1.493	100%	0.0013	0.132
hsa-miR-450b-3p	Primary	2.205	88%	0.0014	0.132
hsa-miR-330-5p	Primary	2.167	85%	0.0016	0.132
hsa-miR-492	Primary	1.802	73%	0.0017	0.132
hsa-miR-452	Primary	2.541	98%	0.0018	0.132
hsa-miR-383	Primary	2.850	98%	0.0026	0.164
hsa-miR-548b	Recurrence	4.178	69%	0.0032	0.168
hsa-miR-382	Primary	1.842	100%	0.0033	0.168
hsa-miR-378	Primary	2.460	85%	0.0035	0.168
hsa-miR-450a	Primary	2.347	98%	0.0040	0.168
hsa-miR-1236	Primary	3.319	75%	0.0041	0.168
hsa-miR-505#	Primary	1.412	96%	0.0045	0.171
hsa-miR-181a-2#	Primary	2.224	96%	0.0060	0.216
hsa-miR-1	Primary	2.416	98%	0.0071	0.232
hsa-miR-625	Primary	1.724	100%	0.0073	0.232
hsa-miR-1253	Primary	1.135	85%	0.0079	0.240
hsa-miR-450b-5p	Primary	1.966	100%	0.0096	0.248
hsa-miR-674	Primary	4.721	83%	0.0096	0.248
hsa-miR-154	Primary	3.068	83%	0.0107	0.248
hsa-miR-185	Primary	1.659	100%	0.0108	0.248
hsa-miR-339-5p	Primary	3.076	96%	0.0109	0.248
hsa-miR-518d-5p	Recurrence	3.008	50%	0.0112	0.248
hsa-let-7a	Primary	1.695	94%	0.0114	0.248
hsa-miR-24-1#	Recurrence	79.356	62%	0.0116	0.248
hsa-miR-124#	Primary	1.521	85%	0.0121	0.249
