# Supplementary material for: MicroRNA expression and DNA methylation profiles do not distinguish between primary and recurrent well-differentiated liposarcoma
Source: PLoS One. 2020 Jan 23;15(1):e0228014. doi: 10.1371/journal.pone.0228014 (PMC6977735; doi:10.1371/journal.pone.0228014)
Supplement: S2 Table — All differentially expressed microRNAs between 15 paired primary and recurrent WDLPS tumor samples of the extremity (p<0.05, FDR<0.25, N = 9 microRNAs) (A) and of the 8 paired primary and recurrent WDLPS tumor samples of the retroperitoneum (p<0.05, no FDR, N = 14 microRNAs)(B). (PDF) [file pone.0228014.s003.pdf]

S2 Table. Differentially expressed microRNAs in subgroup analyses of the extremity and retroperitoneum. All differentially expressed microRNAs between 15 paired primary and recurrent WDLPS tumor samples of the extremity (p<0.05, FDR<0.25, N=9 microRNAs) (**A**) and of the 8 paired primary and recurrent WDLPS tumor samples of the retroperitoneum (p<0.05, no FDR, N=14 microRNAs)(**B**).

(**A**) Extremity					
**microRNA**	**Upregulated in**	**Fold change**	**% detection**	**p-value**	**FDR**
hsa-miR-532-3p	Primary	1.731	100%	0.0001	0.024
hsa-miR-1263	Primary	2.248	63%	0.0001	0.024
hsa-miR-145#	Primary	1.731	100%	0.0008	0.155
hsa-miR-452	Primary	2.609	97%	0.0015	0.221
hsa-miR-100	Primary	1.303	100%	0.0028	0.221
hsa-miR-30d	Primary	1.895	100%	0.0029	0.221
hsa-miR-26b#	Primary	1.422	100%	0.0029	0.221
hsa-miR-33a	Recurrence	1.128	83%	0.0031	0.221
hsa-miR-330-5p	Primary	2.029	80%	0.0034	0.221
(**B**) Retroperitoneum					
**microRNA**	**Upregulated in**	**Fold change**	**% detection**	**p-value**	**FDR**
hsa-miR-30b	Recurrence	1.632	63%	0.0036	0.955
hsa-miR-130b	Recurrence	1.524	88%	0.0051	0.955
hsa-miR-512-3p	Primary	2.444	50%	0.0090	0.955
hsa-miR-340	Recurrence	5.300	88%	0.0090	0.955
hsa-miR-302b#	Recurrence	3.381	69%	0.0144	0.955
hsa-miR-552	Recurrence	1.612	88%	0.0162	0.955
hsa-miR-1304	Recurrence	1.585	81%	0.0201	0.955
hsa-miR-129	Recurrence	1.085	94%	0.0214	0.955
hsa-miR-24-1#	Recurrence	4.316	63%	0.0221	0.955
hsa-miR-130b#	Recurrence	1.476	81%	0.0235	0.955
hsa-miR-136#	Recurrence	1.808	100%	0.0378	0.955
hsa-let-7f	Recurrence	1.884	94%	0.0382	0.955
hsa-miR-885-5p	Primary	2.717	100%	0.0400	0.955
hsa-miR-16-1#	Primary	1.710	81%	0.0483	0.955
