# Supplementary material for: MicroRNA expression and DNA methylation profiles do not distinguish between primary and recurrent well-differentiated liposarcoma
Source: PLoS One. 2020 Jan 23;15(1):e0228014. doi: 10.1371/journal.pone.0228014 (PMC6977735; doi:10.1371/journal.pone.0228014)
Supplement: S3 Table — (PDF) [file pone.0228014.s004.pdf]

S3 Table. List of the numbers of DMRs per chromosome.

Chromosome	Count
chr1	40
chr2	11
chr3	3
chr4	50
chr5	10
chr6	11
chr7	4
chr8	3
chr9	3
chr10	40
chr11	10
chr12	68
chr13	3
chr14	1
chr15	2
chr16	12
chr17	13
chr18	5
chr19	70
chr20	4
chr21	19
chr22	3
chrX	5
chrY	10
No accurate location*	70
**Total**	**470**

*DMRs found in repetitive genomic locations that lack an accurate UCSC chromosomal reference
