# Supplementary material for: MicroRNA expression and DNA methylation profiles do not distinguish between primary and recurrent well-differentiated liposarcoma
Source: PLoS One. 2020 Jan 23;15(1):e0228014. doi: 10.1371/journal.pone.0228014 (PMC6977735; doi:10.1371/journal.pone.0228014)
Supplement: S4 Table — Top 100 genes that contain at least one differentially methylated DNA region (DMR) after Bonferroni correction, excluding genes/DMRs located on the sex chromosomes, found by MeD-seq on 27 paired primary and recurrent WDLPS tumor samples. (PDF) [file pone.0228014.s005.pdf]

S4 Table. Top 100 genes with a DMR. Top 100 genes that contain at least one differentially methylated DNA region (DMR) after Bonferroni correction, excluding genes/DMRs located on the sex chromosomes, found by MeD-seq on 27 paired primary and recurrent WDLPS tumor samples.

No.	Gene	No. of DMRs	Fold Change*	Hypermethylated in	Location
1	RP11-611E13.2	3	2.033	Recurrence	chr12
2	CENPIP1	1	1.661	Primary	chr13
3	FLG-AS1	3	1.590	Primary	chr1
4	HRNR	3	1.590	Primary	chr1
5	MYRFL	3	1.526	Recurrence	chr12
6	RP11-571M6.17	1	1.512	Recurrence	chr12
7	TSFM	2	1.512	Recurrence	chr12
8	AVIL	3	1.512	Recurrence	chr12
9	LYRM4	1	1.376	Recurrence	chr6
10	SLC35E3	5	1.350	Recurrence	chr12
11	OS9	2	1.298	Recurrence	chr12
12	RP11-571M6.7	3	1.298	Recurrence	chr12
13	NOC4L	1	1.293	Primary	chr12
14	MDM2	8	1.293	Recurrence	chr12
15	AC133749.1	1	1.289	Primary	chr12
16	CPM	5	1.289	Primary	chr12
17	CYP27B1	2	1.279	Recurrence	chr12
18	AL671532.6	1	1.278	Recurrence	chr14
19	AC025263.3	3	1.278	Recurrence	chr12
20	MIR26A2	2	1.276	Recurrence	chr12
21	CTDSP2	1	1.276	Recurrence	chr12
22	RP11-159A18.1	1	1.244	Recurrence	chr12
23	AC126281.1	7	1.239	Primary	chr4
24	DUX4L8	6	1.239	Primary	chr4
25	AL671532.5	1	1.239	Recurrence	chr14
26	SHC2	2	1.234	Recurrence	chr19
27	TBC1D22A	1	1.233	Recurrence	chr22
28	RP11-571M6.18	1	1.227	Recurrence	chr12
29	EXOC2	1	1.227	Primary	chr6
30	LRP8	1	1.225	Recurrence	chr1
31	LINC00854	6	1.225	Primary	chr17
32	RP3-470B24.5	1	1.215	Recurrence	chr6
33	AL671532.1	4	1.209	Recurrence	chr14
34	RNA5S9	3	1.203	Primary	chr1
35	AL713899.1	3	1.203	Primary	chr 1
36	GRTP1	1	1.192	Recurrence	chr13
37	SCNN1D	1	1.191	Primary	chr1
38	EXD3	1	1.190	Primary	chr9
39	DUX4L2	6	1.182	Primary	chr4
40	AC126281.4	3	1.182	Primary	chr4

Table S4 continued

No.	Gene	No. of DMRs	Fold Change*	Hypermethylated in	Location
41	AGAP2-AS1	2	1.182	Recurrence	chr12
42	AGAP2	3	1.182	Recurrence	chr12
43	AL845259.5	4	1.178	Primary	chr10
44	DUX4L20	2	1.178	Primary	chr10
45	TSPAN31	3	1.170	Recurrence	chr12
46	CFAP46	1	1.164	Primary	chr10
47	ABCC5	3	1.160	Recurrence	chr3
48	RAB3IP	1	1.157	Recurrence	chr12
49	TCEB3CL2	2	1.153	Recurrence	chr18
50	KATNAL2	3	1.153	Recurrence	chr18
51	AL732375.7	3	1.151	Primary	chr10
52	DIP2C	1	1.150	Primary	chr10
53	PCNT	1	1.146	Primary	chr21
54	DUX4L4	4	1.144	Primary	chr4
55	AC126281.5	3	1.144	Primary	chr4
56	CDK4	2	1.144	Recurrence	chr12
57	RNA5S17	2	1.140	Primary	chr1
58	BEST3	2	1.138	Recurrence	chr12
59	NLRP4	2	1.135	Recurrence	chr19
60	TCEB3CL	3	1.133	Recurrence	chr18
61	TCEB3C	2	1.133	Recurrence	chr18
62	MIR8078	1	1.132	Recurrence	chr18
63	ROCK1P1	1	1.132	Recurrence	chr18
64	ANKRD33B	1	1.114	Recurrence	chr5
65	MARCH9	2	1.114	Primary	chr12
66	CTD-3220F14.1	7	1.113	Primary	chr19
67	METTL21B	2	1.109	Recurrence	chr12
68	RP11-571M6.15	2	1.109	Recurrence	chr12
69	RP11-49K24.9	2	1.109	Recurrence	chr18
70	HMGA2	2	1.109	Recurrence	chr12
71	LMF1	1	1.105	Primary	chr16
72	RP11-611O2.1	1	1.102	Primary	chr12
73	SLC16A3	1	1.102	Recurrence	chr17
74	CSNK1D	1	1.102	Recurrence	chr17
75	RP13-638C3.3	2	1.101	Primary	chr17
76	FOXK2	1	1.101	Primary	chr17
77	YBEY	1	1.100	Primary	chr21
78	AL845259.7	3	1.100	Recurrence	chr10
79	LRRC10	2	1.097	Recurrence	chr12
80	EHMT1	1	1.097	Primary	chr9
81	TMTC2	1	1.092	Primary	chr12
82	TERT	1	1.092	Recurrence	chr5
83	PLEKHG4B	2	1.090	Primary	chr5

Table S4 continued

No.	Gene	No. of DMRs	Fold Change*	Hypermethylated in	Location
84	RP11-620J15.2	1	1.088	Primary	chr12
85	DBET	3	1.087	Primary	chr4
86	RNA5S10	2	1.085	Primary	chr1
87	RNA5S11	2	1.085	Primary	chr1
88	RNA5S12	2	1.085	Primary	chr1
89	RNA5SP19	1	1.082	Primary	chr1
90	RNA5SP162	1	1.082	Primary	chr1
91	DUX4L23	1	1.082	Primary	chr10
92	CTD-3162L10.1	5	1.080	Primary	chr19
93	TMEM242	2	1.078	Recurrence	chr6
94	AL671532.2	1	1.076	Recurrence	chr14
95	DLGAP2	1	1.076	Recurrence	chr8
96	CPSF6	1	1.074	Primary	chr12
97	RNA5S1	2	1.072	Primary	chr1
98	RNA5S2	2	1.072	Primary	chr1
99	RNA5S3	2	1.072	Primary	chr1
100	RNA5S4	3	1.072	Primary	chr1

*Fold change of first/top DMR of the relevant gene
